# S06-4 Exploring correlates of adolescent boys' muscular fitness: a mixed-methods formative study

**DOI:** 10.1093/eurpub/ckac093.031

**Published:** 2022-08-29

**Authors:** Stuart J Fairclough, Laureen Clifford, Lawrence Foweather, Zoe Knowles, Lynne M Boddy, Emma Ashworth, Richard Tyler

**Affiliations:** 1 Movement Behaviours, Nutrition, Health & Wellbeing Research Group, Dept. Sport & Physical Activity, Edge Hill University, UK; 2 Physical Activity Exchange, Research Institute for Sport & Exercise Sciences, Liverpool John Moores University, UK; 3 School of Psychology, Liverpool John Moores University, UK

**Keywords:** Children, stakeholders, motor competence, mental health, participatory

## Abstract

**Background:**

According to the Elaborated Environmental Stress Hypothesis, anxiety and depression in children are associated with poor motor competence, and these associations may be mediated by social support and self-perceptions. Improving children’s motor competence through school-based physical activity interventions may therefore be a mechanism for promoting positive mental health through psychosocial factors. Co-production provides opportunities to participate in intervention development processes, thereby ensuring that specific needs of stakeholders are targeted. The shared stakeholder ownership of the process provides a context-sensitive basis for acceptable interventions with increased likelihood of them being effectively implemented and resulting in positive outcomes. This presentation describes phase 1 of the Move Well, Feel Good study, which aimed to co-produce and evaluate the feasibility of a primary school physical activity intervention to improve children’s motor competence and mental health.

**Methods:**

Five primary schools were recruited from a low socioeconomic status community in northwest England. From these schools, stakeholder groups were formed consisting of class teachers, school leaders, physical activity specialists, and children (aged 8-9 years). Stakeholders worked in single and multiple stakeholder groups through a 6-stage process aligned to the Double Diamond Design Approach by employing divergent and convergent thinking processes to discover, define, develop, and deliver a solution to the ‘problems’ of how best to improve children’s motor competence and mental health, and how best to facilitate real-world school context implementation of the intervention. Through this process the child and adult stakeholders worked separately in workshops and engaged in additional learning and consensus activities. The adult stakeholder co-production workshops were informed by the children’s views and current research evidence. Multiple-stakeholder groups worked collaboratively to develop intervention ideas, which were presented, critiqued, and refined in alignment with the TIDieR checklist.

**Results:**

In the final stage of the process the research team presented the final co-produced interventions back to the stakeholders and a consensus vote was taken to decide which intervention would be implemented in the phase 2 feasibility trial in September 2022.

**Conclusions:**

School stakeholders’ participation in intervention co-production ensures their ownership of the finalised programme, which may be important for subsequent implementation and engagement.

